# Factors associated with and prevalence of depressive features amongst older adults in an urban city in eastern China

**DOI:** 10.4102/sajpsychiatry.v23i0.1064

**Published:** 2017-03-28

**Authors:** Ping Shao, Yong Xu, Chen-Wei Pan

**Affiliations:** 1 School of Public Health, Medical College of Soochow University, China; 2 Department of Health Management, Medical College of Hangzhou Normal University, China

## Abstract

**Background:**

Mental health problems have become serious for older Chinese adults who have lived through the process of urbanisation. This current research aimed to determine the prevalence of and associated factors for depressive features in a community-based sample of older adults in China.

**Methods:**

A community-based survey of 4077 adults aged 60 or older was conducted in Suzhou, China. Information including demographic characteristics, health behaviours, social support, disease histories and physical function was collected using a pre-designed questionnaire. Depressive features were assessed using the self-rating depression scale. Multivariate logistic regression analysis was performed to identify associated factors for depression.

**Results:**

The overall prevalence of depressive features in the surveyed population was 47.4% (45.9% in men and 48.5% in women). In a multiple logistic regression analysis, the significant variables of depressive features were no fixed occupation (odds ratio [OR] = 0.28; 95% confidence interval [CI]: 0.21–0.37), doing non-technical and service work (OR = 0.23; 95% CI: 0.19–0.28) or being a manager and technical personnel (OR = 0.25; 95% CI: 0.19–0.32), physical activities (OR = 0.71; 95% CI: 0.61–0.82), never taking dietary supplements (OR = 0.73; 95% CI: 0.58–0.91), not having hobbies (OR = 1.34; 95% CI: 1.15–1.56), never interacting with neighbours (OR = 1.79; 95% CI: 1.28–2.50), cold relationship with a spouse (OR = 3.34; 95% CI: 1.18–9.45) and limited activities of daily living (OR = 2.27; 95% CI: 1.91–2.69).

**Conclusion:**

There is an urgent need for public policy interventions to address depression in elderly people located in Suzhou in China.

## Introduction

Depression is an important public health concern affecting the health and quality of life amongst the elderly.^1^ Depression may increase the risk of physical and mental diseases (such as cardiovascular and cerebrovascular disease) and the risk of death and other serious consequences. In developed countries, prevalence rates amongst non-institutionalised older people range from 0.4% to 10.2% for depression disorders and from 7.2% to 36.0% for depressive symptoms.^2,3,4^ Depression will become the second highest cause of disease burden by the year 2020.^5^

Risk factors of depression have been widely explored, including socio-demographic factors (gender, age, education level, marital status, economic status, living style),^6,7,8^ health status (sleep disorders, cardiovascular and cerebrovascular diseases, diabetes),^3,9,10^ impaired daily living activities,^6,7,10,11^ cognitive impairment,^7^ living through stressful events,^12^ social support ^4,9,11,13^ and health behaviour.^14^

The process of urbanisation in China has been accelerating in recent years, and the living environment of the elderly has changed dramatically along with this process. Therefore, the mental health of the elderly has been affected in many instances. In meta-analyses, it was found that over time the proportion of older people with depressive symptoms has increased in China.^15^ However, current prevalence estimates are scarce. Studies conducted in mainland China have shown the prevalence of depression symptoms to be 13.01%,^11^ 27.0%,^12^ and 59.8%^16^ amongst older people. Taking into account the complexity and consequences of the problem, it is important to study depressive features of the urban elderly and the associated factors that may have been affected by the process of urbanisation. Suzhou is located in the eastern part of China. Based on a large sample of older people (age 60 +) living in a community in Suzhou, the objectives of this study are as follows:

1. To report the prevalence of depressive features in the elderly.
2. To investigate associated factors with depressive features.

## Methods

This was a community-based study conducted in Suzhou in 2012. A stratified cluster random sampling method was performed, and six districts of Suzhou were divided by socio-economic status into three layers. Three communities in each layer were extracted using a random sampling strategy. People aged 60 or older and living in local areas for more than 5 years were invited to complete a standardised questionnaire. People with cognitive impairments, dementia or disable to communicate with interviewers (such as deaf and mute) were excluded. After investigators explained the goal and the method of the investigation, participants filled out the questionnaires by themselves. The investigators provided detailed explanations to those who had difficulty in understanding the content of the questionnaires. The questionnaires were completed by the investigators for any participants who were illiterate. A total of 4325 questionnaires were issued, and 4077 valid questionnaires were recovered. The response rate was 94.27%.

The Chinese version of Zung’s self-rating depression scale (SDS) was intended to assess depressive features in older people. SDS is a short-range self-rating scale that is easy to operate and which can reflect effectively the severity of depression. It has been widely used, not only amongst the general population, but also specifically for studies involving older populations. In a study of older Brazilians (average age 61.03 years), SDS was validated amongst a group of patients with Parkinson’s diseases. The sensitivity was 88.9%, specificity was 83.3%, the Cronbach coefficient was 0.73 and the SDS and GDS-15 scale scores were highly correlated (*r* = 0.652, *p* < 0.0001).^17^ SDS is composed of 20 items, and total scores range from 20 (best) to 80 (worst). Total scores were divided by 80 to give a final scale ranging from 0.25 to 1. A score of 0.5 or higher is considered as indicating depressive features.

Health behaviours assessed included smoking, drinking, physical exercise and dietary supplement use. Smoking and drinking were differentiated between consumers and non-consumers. With regard to physical exercise, taking exercise 2–3 times per week for 30 min or more was defined as engaging in physical activities.

The number of chronic illnesses was obtained by asking whether a doctor had ever told participants that they had a chronic illness.

Functional ability was measured using the activities of daily living (ADL) scale designed by Elena Yu and William Liu at the University of Illinois at Chicago. The scale’s content was specific, in line with the reality of China, and easy to use. It includes the basic activities of daily living (BADL) scale and instrument activities of daily living (IADL) scale, 20 items in total. Participants were asked about difficulties with performing these activities. Responses were scored using a four-point scale: ‘can do’, ‘there are difficulties’, ‘need help’ and ‘cannot do’. The corresponding score assigned to each response ranged from 1 to 4. Participants with more than two losses of functions or sum scores that were greater than 26 were considered ADL limited. The scale was used amongst 1178 community-dwelling older adults. After 1 year, 1178 cases were retested, and the correlation coefficient was 0.42.^18^

Participants were asked about the frequency of participating in community activities and interacting with neighbours and relatives. In addition, a relationship with a spouse was considered.

Information regarding the following socio-demographic characteristics was also adopted, including gender, age, marital status, income, education and occupation. Occupation referred to the participants’ longest occupation during their lifetimes.

### Data analysis

We used descriptive statistical analysis methods to analyse the basic situation of samples and the distribution of depressive features in the elderly. Pearson’s chi-square test was used to compare the difference of depressive features between the elderly with different characteristics. Significant variables assessed by a chi-square test were included into a binary logistic regression model to analyse the factors that contributed to elderly depressive features. All statistical analyses were conducted using SPSS version 16.0 software (SPSS Inc., Chicago, ILL, USA). All *p*-values less than 0.05 were considered statistically significant.

### Ethical considerations

This study was approved by the Biomedical Research Ethics Committee of the Hangzhou Normal University. All procedures were performed in accordance with the guidelines established by the Declaration of Helsinki. All participants provided written consent before completing the questionnaire.

## Results

### Socio-demographic characteristics and depressive features

The mean age of the participants was 70.6 (s.d.: ± 7.6) years (range 60–99). Amongst the elderly, 81.2% had spouses, 66.9% had an income greater than 1000 RMB per month, 24.5% were illiterate and 34.0% had an education of high school or higher, 26.7% used to be agricultural workers. Of the participants, 53.5% lived with their spouses, whilst 9.2% lived alone (Table 1).

**TABLE 1:** Comparison of socio-demographic characteristics of the elderly by depressive features.

Socio-demographic	Total	Depressive features	χ^2^	*p*
	
	(*n* = 4050), *n* (%)	(*n* = 1904), *n* (%)		
**Sex**				
Male	1814 (44.5)	833 (45.9)	2.7	0.10
Female	2263 (55.5)	1098 (48.5)	-	-
**Age group (years)**				
60~	1120 (27.5)	567 (50.6)	38.3	< 0.001
65~	861 (21.1)	378 (43.9)	-	-
70~	880 (21.5)	375 (42.6)	-	-
75~	659 (16.2)	295 (44.8)	-	-
80~	557 (13.7)	316 (56.7)	-	-
**Monthly income (RMB)**				
< 500	990 (24.3)	753 (76.1)	454.9	< 0.001
500~	360 (8.8)	180 (50.0)	-	-
1000~	2323 (57.0)	847 (36.5)	-	-
2000~	404 (9.9)	151 (37.4)	-	-
**Education**				
Illiterate	997 (24.5)	631 (63.3)	198.7	< 0.001
Elementary	1137 (27.9)	584 (51.4)	-	-
Middle school	966 (23.7)	364 (37.7)	-	-
High school	640 (15.7)	244 (38.1)	-	-
College or above	337 (8.3)	108 (32)	-	-
**Job type**				
Agricultural labour	1089 (26.7)	842 (77.3)	536.3	< 0.001
No fixed occupation	328 (8.0)	130 (39.6)	-	-
Non-technical workers and service workers	2083 (51.1)	752 (36.1)	-	-
Manager and technical personnel	577 (14.2)	207 (35.9)	-	-
**Spouse**				
No	768 (18.8)	421 (54.8)	21.1	< 0.001
Yes	3309 (81.2)	1510 (45.6)	-	-
**Living status**				
Alone	375 (9.2)	205 (54.7)	35.1	< 0.001
Only with spouse	2182 (53.5)	980 (44.9)	-	-
Only with children	947 (23.2)	507 (53.5)	-	-
With spouse and children	573 (14.1)	239 (41.7)	-	-

Significance set at *p* < 0.05. Differences between groups tested with Pearson’s χ^2^ test.

The overall prevalence of depressive features in this population was 47.4% (45.9% in men and 48.5% in women). The prevalence of depressive features was significantly different by age (χ^2^ = 38.3, ***p*** < 0.001), income (χ^2^ = 454.9, ***p*** < 0.001), education level (χ^2^ = 198.7, ***p*** < 0.001), job type (χ^2^ = 536.3, ***p*** < 0.001), spouse (χ^2^ = 21.1, ***p*** < 0.001) and living status (χ^2^ = 35.1, ***p*** < 0.001) (Table 1). Participants over 80 reported the highest frequency of depressive features (56.7%). The proportion of depressive features was higher in those whose monthly income was less than 1000 RMB. Less educated participants, who had an elementary school level of education or lower, had a higher proportion of depressive features than more well-educated participants. Depressive features in participants who were agricultural labourers were more prevalent than in those in the other three job types. Participants with spouses had fewer depressive features (45.6%) than those without a spouse (54.8%). The prevalence of depressive features in elderly people living alone (54.7%) and with their children (53.5%) was higher than those living with a spouse (44.9%) or with a spouse and children (41.7%) (Table 1).

### Health behaviours and depressive features

Depressive features were more prevalent in participants who were considered to be physically inactive when compared with those who were active (57.1% vs. 34.1%, χ^2^ = 211.9, *p* < 0.001). There was a significant association between depressive features and dietary supplement taking (χ^2^ = 45.2, *p* < 0.001). Those who had never taken dietary supplement had the lowest proportion of depressive features (42.7%). Depressive features were more prevalent in older people without hobbies (χ^2^ = 130.2, *p* < 0.001) (Table 2).

**TABLE 2:** Comparison of health behaviours by depressive features.

Health behaviours	Total	Depressive features	χ^2^	*p*
	
	(*n* = 4050), *n* (%)	(*n* = 1904), *n* (%)		
**Smoking**				
No	3323 (81.5)	1564 (47.1)	0.6	0.43
Yes	754 (18.5)	367 (48.7)	-	-
**Alcohol drinking**				
No	3396 (83.3)	1594 (46.9)	1.5	0.22
Yes	681 (16.7)	337 (49.5)	-	-
**Physical activities**				
No	2347 (57.6)	1341 (57.1)	211.9	< 0.001
Yes	1730 (42.4)	590 (34.1)	-	-
**Dietary supplement taking**				
Often	463 (11.3)	203 (43.8)	45.2	< 0.001
Occasionally	1707 (41.9)	914 (53.5)	-	-
Never	1907 (46.8)	814 (42.7)	-	-
**Hobby**				
Yes	1311 (32.2)	451 (34.4)	130.2	< 0.001
No	2766 (67.8)	1480 (53.5)	-	-

Significance set at *p* < 0.05. Differences between groups tested with Pearson’s χ^2^ test.

### Social interaction and family relationships and depressive features

Depressive features were significantly associated with community activities (χ^2^ = 6.2, *p* < 0.001), interacting with relatives (χ^2^ = 22.1, *p* < 0.001) and neighbours (χ^2^ = 11.6, *p* = 0.003) and relationships with a spouse (χ^2^ = 86.7, *p* < 0.001). Participants who often took part in community activities had fewer depressive features (40.8%) than those who occasionally or never took part in community activities (53.2% and 43.6%, respectively). Interacting with neighbours was related to depressive features and the more frequently participants interacted with neighbours, the lower the prevalence of depressive features. Participants with a cold or moderate relationship with their spouse reported higher frequency of depressive features (66.7% and 56.3%, respectively) than those with a close relationship with their spouse (41.8%) (Table 3).

**TABLE 3:** Comparison of social interaction and family relationship of the elderly by depressive features.

Characteristics	Total	Depressive features	χ^2^	*p*
	
	(*n* = 4050), *n* (%)	(*n* = 1904), *n* (%)		
**Participating in community activities**				
Often	762 (18.7)	311 (40.8)	46.2	< 0.001
Occasionally	1814 (44.5)	965 (53.2)	-	-
Never	1501 (36.8)	655 (43.6)	-	-
**Interacting with relatives**				
Often	2302 (56.5)	1022 (44.4)	22.1	< 0.001
Occasionally	1625 (39.9)	843 (51.9)	-	-
Never	150 (3.6)	66 (44)	-	-
**Interacting with neighbours**				
Often	2243 (55)	1015 (45.3)	11.6	0.003
Occasionally	1666 (40.9)	822 (49.3)	-	-
Never	168 (4.1)	94 (56)	-	-
**Relationship with spouse**				
Close	2456 (60.2)	1026 (41.8)	86.7	< 0.001
Moderate	1299 (31.9)	731 (56.3)	-	-
Cold	19 (0.4)	13 (66.7)	-	-
Difficult evaluating	35 (0.8)	15 (42.9)	-	-

Significance set at *p* < 0.05. Differences between groups tested with Pearson’s χ^2^ test.

### Health status and physical function and depressive features

Depressive features in the elderly with limited ADL were more prevalent than in the elderly with a normal ADL (71.2% vs. 39.7%, χ^2^ = 299.1, *p* < 0.001) (Table 4).

**TABLE 4:** Comparison of health status and physical function of the elderly by depressive features.

Characteristics	Total	Depressive features	χ^2^	*p*
	
	(*n* = 4050), *n* (%)	(*n* = 1904), *n* (%)		
**Number of chronic diseases**				
0	1277 (31.3)	609 (47.7)	3.9	0.28
1	1629 (40)	773 (47.5)	-	-
2	788 (19.3)	354 (44.9)	-	-
≥ 3	383 (9.4)	195 (50.9)	-	-
**ADL**				
Normal	3084 (75.6)	1224 (39.7)	299.1	< 0.001
Limited	993 (24.5)	707 (71.2)	-	-

Significance set at *p* < 0.05. Differences between groups tested with Pearson’s χ^2^ test.

### Associated factors of depressive features

The result of multiple logistic regression analyses showed that significant variables of depressive features were job type, physical activity, taking dietary supplements, interacting with neighbours, relationship with spouses, hobbies and ADL (  *p* < 0.05). Compared with agricultural labourers, older people without fixed occupations, non-technical and service workers, and managers and technical personnel had fewer depressive features. Those participating in physical activity were 0.71 times less likely to be depressed than those without physical exercises (  *p* < 0.001). Compared with participants who often took dietary supplement, those who never took dietary supplements had fewer depressive features (  *p* = 0.005). Older people without hobbies were 1.34 times more likely to be depressed than those with hobbies (  *p* < 0.001). Older people who never interacted with neighbours were 1.79 times more likely to be depressed than those often interacting with their neighbours (  *p* = 0.001). Participants who had cold relationships with spouses were 3.34 times more likely to be depressed than those with close relationships with spouses (  *p* = 0.023). Those with limited ADL were 2.27 times more likely to be depressed than those with a normal ADL (  *p* < 0.001) (Table 5).

**TABLE 5:** Logistic regression model of the elderly on depressive features.

Independent variables	Coefficient	S.E.	Wald	*p*	OR	95% CI of OR	

						Lower	Upper
**Job type (reference: agricultural labour)**			**238.99**	**< 0.001**			
No fixed occupation	−1.29	0.15	79.16	< 0.001	0.28	0.21	0.37
Non-technical workers and service workers	−1.46	0.10	232.31	< 0.001	0.23	0.19	0.28
Manager and technical personnel	−1.39	0.13	123.45	< 0.001	0.25	0.19	0.32
**Physical activities(reference: no)**				**< 0.001**			
Yes	−0.35	0.08	21.12	< 0.001	0.71	0.61	0.82
**Dietary supplement taking (reference: often)**			**20.44**	**< 0.001**			
Occasionally	−0.002	0.12	0.001	0.98	1.01	0.80	1.26
Never	−0.32	0.11	7.76	0.005	0.73	0.58	0.91
**Hobby (reference: yes)**							
No	0.29	0.08	14.33	< 0.001	1.34	1.15	1.56
**Interacting with neighbours (reference: often)**			**15.76**	**< 0.001**			
Occasionally	−0.10	0.08	1.74	0.19	0.90	0.78	1.05
Never	0.58	0.17	11.52	0.001	1.79	1.28	2.50
**Relationship with spouse (reference: close)**			**20.69**	**0.001**			
Moderate	0.13	0.08	2.44	0.12	1.14	0.97	1.34
Cold	1.21	0.53	5.19	0.023	3.34	1.18	9.45
Difficult evaluating	−0.04	0.36	0.01	0.92	0.97	0.48	1.97
**ADL (reference: normal)**							
Limited	0.82	0.09	86.31	< 0.001	2.27	1.91	2.69
Constant	0.81	0.15	28.32	< 0.001	2.23	-	-

Significance set at *p* < 0.05.

S.E., standard error; OR, odds ratio; 95% CI, 95% confidence interval.

## Discussion

The survey results showed that the prevalence of depressive features in the elderly was 47.4% (45.9% in male and 48.5% in women). These results are higher than those observed in India (39.1%),^19^ in Hong Kong (30.0%),^20^ amongst elderly Chinese immigrants in Western countries (20.0% – 30.0%),^21^ and in South Korea (26.0%),^22^ as well as being lower than that of elderly Chinese (59.8%)^16^ reported in previous studies. The differences may be due to a difference in sampling design, assessment tools for depressive features or objects of study.

This study was conducted in an urban area, but a number of participants in the investigation had lived in the countryside in the past. In the process of urbanisation, these rural communities gradually transformed into urban communities, so the longest occupation for a considerable part of the investigated population was agricultural labour. The study found that the longest occupation in a participant’s career was associated with depressive features. Compared with the agricultural labour group, older people without a fixed occupation, those in non-technical and service work, and management or technical personnel had lower rates of depressive features. This indicates that, in the process of urbanisation, participants who used to be farmers had a greater risk of depressive features. A study focused on the elderly in Beijing found that depressive symptoms in the elderly in rural areas were higher than in similar urban populations.^11^ It showed that the prevalence of depressive symptoms in rural and urban elderly populations was 26.63% and 10.79%, respectively. Depressive symptoms amongst rural elderly were significantly more prevalent than in the urban elderly. This was similar to our results.

Regular physical exercise can reduce the risk of depression in the elderly.^23,24^ This observation has been confirmed in our study. Elderly who never take dietary supplements had a lower risk of depressive features than those who took dietary supplements often (odds ratio [OR] = 0.73, 95% confidence interval [CI]: 0.58–0.91). This may be because the elderly who never take dietary supplements are healthier and do not think they need dietary supplements. Good health status was a protective factor of depression.^3^ Hobbies can help enrich amateur life and regulate life pressure, and it was good for mental health; those who had a hobby also showed a lower risk of depressive features.

This study showed that neighbourhood interaction and intimate marital relationships were protective factors of depression. A large number of studies have shown that social support has an important impact on mental health and social interaction between people can provide more supportive interaction.^25^ Active social relationships and support help the elderly relieve the adverse effects stressful events have on health. It can also encourage the elderly to seek prevention or appropriate medical treatment and better adhere to medication or treatment plan, and become less involved in unhealthy or negative behaviours.^26,27^ These actions can better promote health. Lack of social interaction and support will result in social isolation, increasing loneliness, formation of the subjective sense of social isolation and increase the risk of depression in elderly populations.^13^ The results here also indicate that not all elderly who have a spouse (81.2%) lived with their spouse only. Considering the convenience of taking care of grandchildren and other reasons, some of the elderly lived with extended family as well or were separated from their spouses. However, living arrangement was not found to be a significant factor. This may be due to the impact of other factors. The results showed intimate marital relationship is a protective factor of depressive features. This study showed that the risk of depressive features for ADL-limited participants was 2.27 times greater than the normal elderly. Older adults with limited ADL tended to have negative emotions. Physical and mental health would be adversely affected. Several studies have found that decline of normal abilities in daily life significantly increased the risk of depression in the elderly, which is similar to the results of this study.^6,28^

The present results must be considered within the following limitations. This was a cross-sectional study, which cannot suggest causation between exposures and outcomes. Furthermore, information on living through stressful events was not collected and should be included in further investigations.

## Conclusion

The elderly in Suzhou suffering from depressive features are in urgent need of intervention via public health policies. In our study, we found that occupation, physical activity, taking dietary supplements, neighbour interaction, marital relationship, hobbies and ADL were associated factors of depressive features. These findings will contribute to the development of elderly depression interventions in public health policy.

## References

[1] Sivertsen H, Bjorklof GH, Engedal K, Selbaek G, Helvik AS Depression and quality of life in older persons: A review. Dement Geriatr Cogn Disord. 2015;40(5):311–339. 26360014 10.1159/000437299

[2] Bojorquez-Chapela I, Villalobos-Daniel VE, Manrique-Espinoza BS, Tellez-Rojo MM, Salinas-Rodríguez A Depressive symptoms among poor older adults in Mexico: Prevalence and associated factors. Rev Panam Salud Publ. 2009;26(1):70–77. 19814885

[3] Chang-Quan H, Xue-Mei Z, Bi-Rong D, Zhen-Chan L, Ji-Rong Y, Qing-Xiu L Health status and risk for depression among the elderly: A meta-analysis of published literature. Age Ageing. 2010;39(1):23–30. 10.1093/ageing/afp187 19903775

[4] Fiske A, Wetherell JL, Gatz M Depression in older adults. Annu Rev Clin Psychol. 2009;5:363–389. 10.1146/annurev.clinpsy.032408.153621 19327033 PMC2852580

[5] Bongongo T, Tumbo J, Govender I Depressive features among adult patients receiving antiretroviral therapy for HIV in Rustenburg district, SA. S Afr J Psych. 2013;19(2):31–34. 10.7196/sajp.418

[6] Érico CC, Maria FL, Sandra C, Josélia OF, Uchoa E Factors associated with depressive symptoms measured by the 12-item General Health Questionnaire in Community-Dwelling Older Adults (The Bambul Health Aging Study). Rev Bras Psiquiatr. 2008;30(2):104–109. 10.1590/S1516-44462008005000007 18470408

[7] Weyerer S, Eifflaender-Gorfer S, Kohler L, et al Prevalence and risk factors for depression in non-demented primary care attenders aged 75 years and older. J Affect Disord. 2008;111(2):153–163. 10.1016/j.jad.2008.02.008 18372049

[8] Gibson RC, Neita SM, Abel WD, James K, Eldemire-Shearer D Sociodemographic factors associated with depressive symptoms among elderly persons from two communities in Kingston, Jamaica. West Indian Med J. 2013;62(7):615–619. 10.7727/wimj.2012.273 24831899

[9] Chan MF, Zeng W Investigating factors associated with depression of older women in Macau. J Clin Nurs. 2009;18(21):2969–2977. 10.1111/j.1365-2702.2009.02867.x 19732244

[10] Peltzer K, Phaswana-Mafuya N Depression and associated factors in older adults in South Africa. Glob Health Action. 2013;6:1–9. 10.3402/gha.v6i0.18871 PMC3549465 23336621

[11] Li N, Pang L, Chen G, Song X, Zhang J, Zheng X Risk factors for depression in older adults in Beijing. Can J Psychiatry. 2011;56(8):466–473. 10.1177/070674371105600804 21878157

[12] Yunming L, Changsheng C, Haibo T, et al Prevalence and risk factors for depression in older people in Xi’an China: A community-based study. Int J Geriatr Psychiatry. 2012;27(1):31–39. 10.1002/gps.2685 21284042

[13] Coyle CE, Dugan E Social isolation, loneliness and health among older adults. J Aging Health. 2012;24(8):1346–1363. 23006425 10.1177/0898264312460275

[14] Bots S, Tijhuis M, Giampaoli S, Kromhout D, Nissinen A Lifestyle- and diet-related factors in late-life depression – A 5-year follow-up of elderly European men: The FINE study. Int J Geriatr Psychiatry. 2008;23(5):478–484. 10.1002/gps.1919 17975846

[15] Li D, Zhang DJ, Shao JJ, Qi XD, Tian L A meta-analysis of the prevalence of depressive symptoms in Chinese older adults. Arch Gerontol Geriatr. 2014;58(1):1–9. 10.1016/j.archger.2013.07.016 24001674

[16] Hu LR, Zheng CJ, Zhang GH The elderly community residents quality of life and depression. Chin J Gerontol. 2015;19(3):32–35. (In Chinese)

[17] Chagas MH, Tumas V, Loureiro SR, et al Validity of a Brazilian version of the Zung self-rating depression scale for screening of depression in patients with Parkinson’s disease. Parkinsonism Relat Disord. 2010;16(1):42–45. 10.1016/j.parkreldis.2009.07.010 19660977

[18] Zhang M Study on nutritional status and related factors of patients with dysphagia in senile dementia [PhD thesis]. Taishan Medical University, China, Tai’an; 2013 (In Chinese)

[19] Nakulan A, Sumesh TP, Kumar S, Rejani PP, Shaji KS Prevalence and risk factors for depression among community resident older people in Kerala. Indian J Psychiatry. 2015;57(3):262–266. 26600579 10.4103/0019-5545.166640 PMC4623644

[20] Chan WC, Kwan CW, Chi I Moderating effect of communication difficulty on the relationship between depression and pain: A study on community-dwelling older adults in Hong Kong. Aging Ment Health. 2015;19(9):829–834. 10.1080/13607863.2014.967172 25316214

[21] Lin X, Haralambous B, Pachana NA, et al. Screening for depression and anxiety among older Chinese immigrants living in Western countries: The use of the Geriatric Depression Scale (GDS) and the Geriatric Anxiety Inventory (GAI). Asia Pac Psychiatry. 2016;8(1):32–43. 10.1111/appy.12191 26010903

[22] Kim BS, Lee DW, Bae JN, et al. Impact of illiteracy on depression symptomatology in community-dwelling older adults. Int Psychogeriatr. 2014;26(10):1669–1678. 10.1017/S1041610214001094 24945628

[23] Tsai AC, Chi SH, Wang JY Cross-sectional and longitudinal associations of lifestyle factors with depressive symptoms in ≥ 53-year old Taiwanese – Results of an 8-year cohort study. Prev Med. 2013;57(2):92–97. 10.1016/j.ypmed.2013.04.021 23651861

[24] Paulo TRS, Tribess S, Sasaki JE, et al. A cross-sectional study of the relationship of physical activity with depression and cognitive deficit in older adults. J Aging Phys Act. 2016;24(2):311–321. 10.1123/japa.2014-0253 26439455

[25] Teo AR, Choi H, Andrea SB, et al. Does mode of contact with different types of social relationships predict depression in older adults? Evidence from a nationally representative survey. J Am Geriatr Soc. 2015;63(10):2014–2022. 10.1111/jgs.13667 26437566 PMC5527991

[26] Kawachi I, Berkman LF Social ties and mental health. J Urban Health. 2001;78(3):458–467. 10.1093/jurban/78.3.458 11564849 PMC3455910

[27] Hughes ME, Waite LJ Health in household context: Living arrangements and health in late middle age. J Health Soc Behav. 2002;43(1):1–21. 10.2307/3090242 11949193 PMC1440422

[28] Russo A, Cesari M, Onder G, et al. Depression and physical function: Results from the aging and longevity study in the Sirente geographic area (ilSIRENTE Study). J Geriatr Psychiatry Neurol. 2007;20(3):131–137. 10.1177/0891988707301865 17712095

